# The Value of the Antibody Detection in the Diagnosis of Ocular Toxocariasis and the Aqueous Cytokine Profile Associated With the Condition

**DOI:** 10.3389/fmed.2022.838800

**Published:** 2022-03-28

**Authors:** Xiang Zhang, Yuan Yang, Yan Zheng, Yiqian Hu, Yuqing Rao, Jiakai Li, Peiquan Zhao, Jing Li

**Affiliations:** Department of Ophthalmology, Xinhua Hospital Affiliated to Shanghai Jiao Tong University School of Medicine, Shanghai, China

**Keywords:** ocular toxocariasis, diagnosis, aqueous humor, antibody detection, Th2 response, cytokines

## Abstract

**Introduction:**

To evaluate and compare the specificity of Toxocara *canis*-specific antibody detection in the serum and aqueous samples for the diagnosis of ocular toxocariasis (OT) and explore the cytokine profiles associated with the condition in children.

**Materials and Methods:**

This is a prospective cohort study. The inclusion criteria were the clinical presentations of OT, which included unilateral vision reduction, typical peripheral or posterior pole granuloma with variable degrees of vitritis, and exclusion of other diagnoses. The titer of antibody against the excretory-secretory antigen of Toxocara *canis* [T-immunoglobulin G (IgG)] was measured in serum and aqueous samples that were taken from the affected eyes. The diagnosis of OT was made upon positive detection of T-IgG either in the serum or aqueous. The rest with typical clinical presentations as described above but a positive serum or aqueous T-IgG could not be confirmed were diagnosed as suspected OT. Cytokines were measured using multiplexed cytometric bead array system.

**Results:**

Two hundred and eleven eyes of 211 patients had participated in the study. One hundred and twenty-eight eyes were diagnosed as OT. The median age of the cohort was 7.7 years with a male to female ratio of 2.5:1. Major initial symptoms were decreased vision (74%) and strabismus (22%). The percentages of eyes with peripheral granuloma, posterior granuloma, and endophthalmitis were 40, 18, and 41%, respectively. Vitritis (100%), vitreous strands (64%), retinal fibrotic bands (57%), and retinal detachment (42%) were the most common signs. T-IgG was positive in 66.7% of the aqueous and 57.2% of the serum samples. Forty-four patients were diagnosed T-IgG negative in both serum and aqueous of the affected eyes. Interleukin (IL)-6, monocyte chemoattractant protein (MCP)-1, IL-8, eosinophil chemotactic protein (Eotaxin), MCP-1β, and vascular endothelial growth factor (VEGF) were higher in T-IgG negative eyes when compared to controls and further increased in T-IgG positive eyes. However, only T-IgG positive eyes showed increased IL-5, IL-13, and IL-10. IL-1β, tumor necrosis factor-alpha (TNF-α), IL-12, IL-2, interferon-gamma (IFN-γ), and IL-4 were undetectable in all eyes.

**Conclusions:**

Pediatric OT is often present with severe retinal complications. Polarized intraocular Th2 response was only found in aqueous T-IgG positive eyes. Our results supported an aqueous sample-based antibody test for the more specific diagnosis of OT.

## Introduction

Ocular toxocariasis (OT) is a condition caused by the infection of roundworms, mainly *Toxocara canis*, less frequently by *Toxocara cati* and other helminth species in the eyes (1, 2). Humans become infected through unintentional ingestion of the infective eggs. The eggs hatch in the digestive tract, penetrate the intestine, and spread *via* circulation. The larva is not able to mature within the human body and instead encysts in tissues. Migrating, dying, or dead larva could stimulate eosinophilic responses in host tissues. Increased number of eosinophils, increased concentrations of total immunoglobulin E (IgE), and increased levels of interleukin (IL)-4, IL-6, IL-5, IL-10, IL-13, and interferon-gamma (IFN-γ) were reported in peripheral blood of patients with systemic signs of Toxocara infection (3, 4). However, there was a paucity of data on intraocular immunological responses associated with OT.

The human eye is one of the organs that the larva prefers to stay. However, the diagnosis of OT could be challenging due to the lack of pathognomonic signs. The main symptoms of OT include decreased vision, strabismus, and leukocoria. OT can manifest in three major types (5): 1. Peripheral granulomatous type, which is featured by a focal, increased, white nodule granuloma located at the peripheral retina. 2. Posterior granulomatous type, which is featured by granuloma at the posterior pole. 3. Chronic endophthalmitis type, which is featured by diffuse intraocular inflammation, often more severe in the vitreous cavity than in the anterior chamber. The granulomatous types appeared to be the dominant types of OT manifestation, often accounting for more than 70% of the affected eyes (6, 7). However, endophthalmitis type may be more frequently seen in children, as reported by an early study from Poland (8). Other clinical signs include vitreous bands, epiretinal membrane, fibrotic retinal bands, retinal folds, partial, or total retinal detachment. Posterior synechiae and signs of inflammation in the anterior chamber are less frequently seen (7). The antibodies against excretory-secretory proteins of *T. canis* [T-immunoglobulin G (T-IgG)] in serum or intraocular fluid are often measured to aid the diagnosis of OT (6). However, many studies have reported negative T-IgG in serum or aqueous samples taken from clinically diagnosed OT patients (7, 9–14). These patients were clinically undistinguishable from the T-IgG positive patients. Whether there were differences in the underlying immunological responses between T-IgG positive and negative eyes were unknown.

Due to the nature of the infection, Toxocara infection is more often seen in children than in adults. Toxocara seroprevalence ranged from 4 to 46% in adults and can be as high as 77.6% in school children (9, 15, 16). The reported seroprevalence among children in China varied from 5.14 to 19.3% (17–19). Data on the prevalence of OT were scarce, the reported prevalence varied from 1 case per 1,000 persons in the general population to 7 ophthalmologist-diagnosed OT cases in 100,000 school children (20, 21). OT is one of the main causes of uveitis and blindness in children. Since children are less likely to notice and report changes in vision acuity, the symptoms and clinical signs of pediatric OT could be more complicated than the adult version. In this prospective study, we characterized the clinical and immunological features of 211 consecutive pediatric cases seen in a tertiary ophthalmology clinic from June 2014 to June 2018. Their T-IgG was measured in both aqueous and serum samples and the association between intraocular cytokine responses and T-IgG status was explored.

## Materials and Methods

### Patients and Ethical Statement

This is a prospective study carried out at the Department of Ophthalmology, Xinhua Hospital affiliated to Shanghai Jiao Tong University School of Medicine between June 2014 and June 2018. In total, 128 consecutive pediatric patients (1–18 years old) who were diagnosed with OT, and 83 pediatric patients who were presented with unilateral retinal granuloma or vitritis without explainable causes but a positive serum or aqueous T-IgG could not be confirmed were enrolled upon consent by their parent(s) or legal guardian(s). Twenty-five children with congenital cataracts but otherwise quiescent eyes who underwent cataract surgery at the same time period were recruited and their aqueous samples were used as controls for cytokine analysis as described below. The study protocol was approved by the Institutional Review Board of Xinhua Hospital and adhered to the tenets of the Declaration of Helsinki. All examinations and procedures were performed in accordance with the approved protocol. Informed written consent was obtained from the parents/guardians of the patients.

### Patient Evaluation and the Diagnostic Criteria for OT

For all enrolled patients, the medical history and information about the patient's living environment and previous contact with animals, especially dogs, were obtained. The ophthalmic examinations included vision acuity test, intraocular pressure measurement, slit-lamp examination, B-scan ultrasonography, and funduscopic examinations. Depending on the conditions of the eyes, ultrasound biomicroscopy (UBM) (SW-3200, Suoer, Tianjin, China), scanning laser ophthalmoscopy (Optomap 200Tx; Optos, Inc., Marlborough, MA, USA), optical coherence tomography (RTVue XR AVANTI with AngioVue; Optovue Inc., Fremont, CA, USA), and fundus fluorescein angiography (HRA2; Heidelberg Engineering GmbH, Heidelberg, Germany) were also performed. For children <3-year old, RetCam imaging (RetCam-120 digital retinal camera; Clarity Medical Systems, Pleasanton, CA, USA) was performed.

Ocular toxocariasis was diagnosed by the same senior retina specialist based on the following criteria: 1. Unilateral vision reduction, strabismus, leukocoria, red or painful eye, or photophobia. 2. The existence of peripheral or posterior pole granuloma with variable degrees of vitritis or moderate to severe vitritis with vitreous strands, or layered vitreous veil or retinal fibrosis. 3. Positive serum or aqueous T-IgG (see next section for details). The severity of vitritis was graded as mild, moderate, and severe based on vitreous haze according to the grading system proposed by the National Eye Institute and agreed by two doctors (22). The suspected cases were those with typical clinical presentations, such as OT, as described above but a positive serum or aqueous T-IgG could not be confirmed.

For all patients, the following conditions were excluded: retinoblastoma, toxoplasmic retinochoroiditis, retinopathy of prematurity (ROP), familial exudative vitreoretinopathy (FEVR), persistent fetal vasculature, Coats' disease, ocular tuberculosis, syphilis, retinitis, and organized vitreous hemorrhage.

### T-IgG and Total IgG Measurement and the Calculation of Goldmann-Witmer Co-efficient

Aqueous samples were taken under sterile conditions using a 30-gauge needle on a tuberculin syringe *via* an anterior chamber paracentesis as previously described (23). T-IgG was measured using a qualitative ELISA kit (*Toxocara canis* IgG ELISA, IBL International, Hamburg, Germany). The specificity and sensitivity were both above 95%. Serum and aqueous samples were 100 times diluted and analyzed in duplicates. The titer of the antibody was calculated as follows: (absorbance of sample ×10)/absorbance of cutoff control. According to the manufacturer's instructions, a serum sample with a titer of <9 units was determined as negative, higher than 11 was positive, between 9 and 11 was uncertain. Since the manufacturer did not provide a cutoff value for aqueous samples, we measured T-IgG in 25 aqueous samples that were taken from patients with no clinical signs of OT or uveitis in general. The mean titer was 0.880, with a range between 0.000 and 2.953 and a standard deviation (SD) of 0.677. Based on these values, we set a cutoff value of 2.234 (mean + 2 SD) for aqueous samples.

To calculate GWC, total IgG levels in serum and aqueous samples were quantitated using an ELISA kit (Abnova, KA3976). Serum and aqueous samples were 5,000 and 50 times diluted, respectively, and analyzed in duplicates. GWC was calculated as follows: (T-IgG/total IgG) aqueous/(T-IgG/total IgG) serum.

### Cytokine Analysis

Bio-Plex Pro^TM^ magnetic color-bead-based multiplex assay (Bio-Rad Laboratories, Inc., Hercules, CA, USA) was used to measure the concentrations of the following human cytokines: IL-1β, IL-2, IL-4, IL-5, IL-6, IL-8, IL-10, IL-12(p70), IL-13, IL-17, IFN-γ, tumor necrosis factor-alpha (TNF-α), monocyte chemoattractant protein (MCP-1, CCL2), macrophage inflammatory protein-1α (MIP-1α, CCL3), macrophage inflammatory protein-1β (MIP-1β, CCL4), regulated on activation normal T cell expressed and secreted (RANTES, CCL5), eosinophil chemotactic protein (Eotaxin, CCL11), granulocyte-colony stimulating factor (GCSF), and vascular endothelial growth factor (VEGF). The assay was performed according to the manufacturer's instructions and analyzed using Bio-Plex^TM^ 200 System (software version 6.0). Fifty microliters (50 μl) of aqueous humor sample were used in each reaction. All concentrations that were determined as < out of range (OOR) by the software were arbitrarily determined as 0 for data analysis.

### Statistical Analysis

Statistical analysis was conducted using Statistical Package for the Social Sciences (SPSS) Version 19 (IBM Corporation, Armonk, NY, USA). We used the unpaired *t*-test or the Mann-Whitney U test to compare the differences in cytokines between 2 groups, depending on the distribution of data. A significance value of or < 0.05 was accepted as statistically significant.

## Results

### Demographic Information of Patients Involved in the Study

Two hundred and eleven consecutive pediatric patients with typical clinical presentation of OT were enrolled. One hundred and twenty-eight were diagnosed with OT since their serum or aqueous was T-IgG positive. The rest were diagnosed as suspected OT. Among those, 44 were confirmed both serum and aqueous T-IgG negative.

The demographic information of all 211 patients is summarized in Table 1. The male to female ratio was 2.5:1. The median age of patients at the time of diagnosis was 7.68 years. There was no significant difference in age between male and female patients. About 46.4% of the patients had lived in the rural areas (villages), 20.4% lived in a town or city, 33.2% had lived in both village and town areas before the onset of the symptoms. Most of the patients had gone to other ophthalmology clinics before coming to us. About 74.4% of the patients had initial signs of reduced vision, 22.3% had strabismus, 7.1% had leukocoria. The median time from the notice of the symptoms to the diagnosis of OT was 2 months, with a range of 3 days to 6 years.

**Table 1:** Demographic information and initial symptoms.

No. of patients	211
Gender (No. of patients, % in the cohort)	
Male	151 (70.9%)
Female	60 (28.2%)
Age (Median and range, in years)	7.7 (1–18)
Time till diagnosis (Median and IQR, in days)	60 (3->3 yrs)
Living environment (No. of patients, % in the cohort)	
Town or city	43 (20.4%)
Rural area	98 (46.4%)
Mixed	70 (33.2%)
History of dog contact (No. of patients, % in the cohort)	
Yes	158 (74.9%)
No	46 (21.8%)
Initial symptoms (No. of patients, % in the cohort)	
Decreased vision	157 (74.4%)
Strabismus	47 (22.3%)
Leukocoria	15 (7.1%)
Red eye	8 (3.8%)
Pain	3 (1.4%)
Photophobia	2 (1.0%)

### Ophthalmic Findings

**Figure 1:**
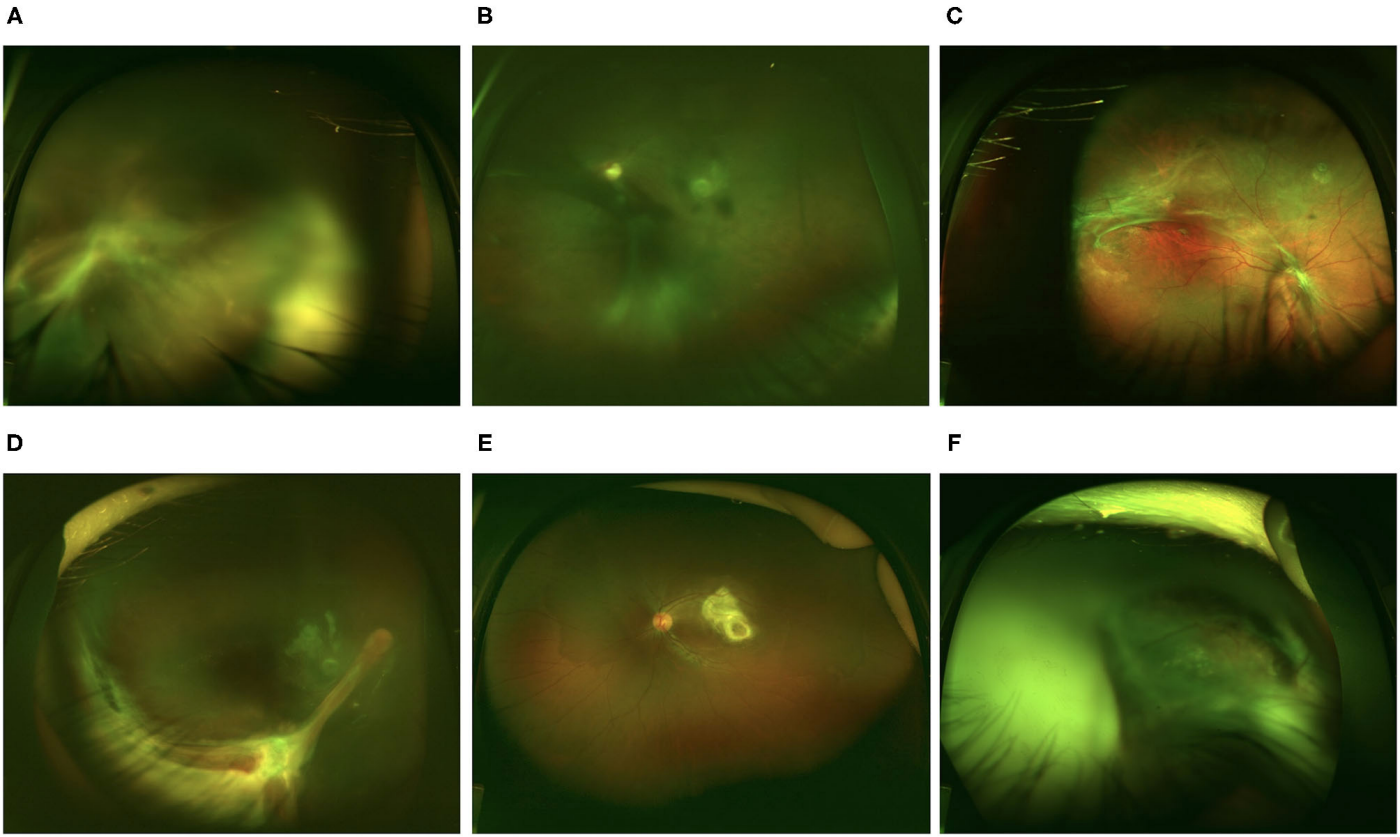
Representative retinal images of eyes with ocular toxocariasis (OT). **(A–C)** Endophthalmitis type OT. **(A)** Diffusive vitritis with vitreous strands extending to the retina. **(B)** Diffusive vitritis with vitreous membrane. **(C)** Fibrotic membrane at the retina surface with relatively clear vitreous. **(D)** Peripheral granuloma with retinal fold extending to the optic disc. **(E)** Posterior pole granuloma. **(F)** Total retinal detachment with vitritis.

The entire cohort was separated into three conventional groups based on clinical presentations, and the major ophthalmic findings are summarized in Table 2. Four eyes from 4 patients had both peripheral and posterior granulomas. They were not included because we were not sure which group they belong to. Therefore, 207 eyes from the original 211 patients were further analyzed. In total, 86 eyes (41.5%) were of the endophthalmitis type, 38 eyes (18.4%) and 83 eyes (40.1%) were of the posterior and peripheral granuloma types, respectively. Typical clinical presentations of the affected eyes are shown in Figure 1.

**Table 2:** Clinical features.

**Variables**	**No. of eyes (% in the group)**			
	**Peripheral granuloma**	**Posterior granuloma**	**Endophthal-mitis**	**Overall**
No. of eyes	83 (40.1%)	38 (18.4%)	86 (41.5%)	207 (100%)
Vitritis				
Severe	26 (31.3%)	6 (15.8%)	49 (57.0%)	82 (38.9%)
Moderate	26 (31.3%)	11 (28.9%)	25 (29.1%)	63 (30.0%)
Mild	31 (37.3%)	21 (55.3%)	12 (14.0%)	66 (31.3%)
Vitreous strands	59 (71.1%)	18 (47.4%)	54 (62.8%)	134 (63.5%)
Vitreous veil	7 (8.4%)	0	9 (10.5%)	17 (8.1%)
Retinal fibrotic band	56 (67.5%)	32 (84.2%)	29 (33.7%)	120 (56.9%)
Dragged disc	29 (34.9%)	18 (47.4%)	10 (11.6%)	58 (27.5%)
Epiretinal membrane	8 (9.6%)	10 (26.3%)	9 (10.5%)	29 (13.7%)
Retinal detachment				
Partial	24 (28.9%)	7 (18.4%)	7 (8.1%)	39 (18.5%)
Total	13 (15.7%)	3 (7.9%)	35 (40.7%)	50 (23.7%)
Cyclitic membrane	2 (2.4%)	0	2 (2.3%)	4 (1.9%)
Posterior synechiae	7 (8.4%)	2 (5.3%)	16 (18.6%)	25 (11.8%)
Cataract	8 (9.6%)	0	8 (9.3%)	16 (7.6%)
Anterior KP	10 (12.0%)	4 (10.5%)	10 (11.6%)	25 (11.8%)
Band keratopathy	8 (9.6%)	1 (2.6%)	8 (9.3%)	17 (8.1%)

There were significant differences in the severity of vitritis among three types (Kruskal-Wallis analysis, *p* < 0.001). Severe vitritis was most frequently found in the endophthalmitis type (57%). There were 12 endophthalmitis-type eyes with mild vitritis; however, they all had either vitreous bands or retinal fibrosis without other explainable causes. The endophthalmitis type also had a higher percentage of posterior synechiae (18.6%) than other types. The occurrence of anterior keratic precipitates (KP) and band keratopathy was similar among the three groups.

Fibrotic changes of the vitreous and retina were common. Vitreous strands were found in 71.1, 47.4, and 62.8% of the peripheral granuloma, posterior granuloma, and endophthalmitis types, respectively. Retinal fibrotic changes were found in 67.5, 84.2, and 33.7% of the peripheral granuloma, posterior granuloma, and endophthalmitis types, respectively. The posterior granuloma type had the highest percentage of eyes with optic disc involvement (47.4%) and the epiretinal membrane (26.3%).

Within the entire cohort, about 42% of eyes had varying degrees of retinal detachment. In the granulomatous types, the partial detachment was more frequently seen than complete detachment. However, in the endophthalmitis type, 48.8% of eyes had retinal detachment and 83.3% of those had complete detachment.

Other less frequently seen clinical signs included cataracts, which occurred in <10% of eyes. Since most patients were too young to cooperate with UBM examinations, cyclitic membrane was found in about 2% of eyes during vitrectomy.

### Aqueous and Serum T-IgG

**Figure 2:**
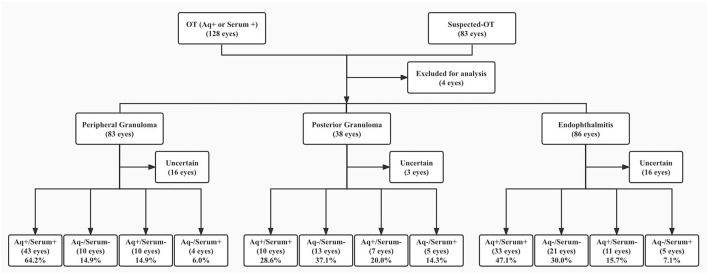
Patient groups according to clinical features and aqueous and serum T-IgG status. T-IgG status referred to positive, negative, or uncertain of the anti-*T. canis* antibody in samples. + referred to T-IgG positive, – referred to T-IgG negative. OT, ocular toxocariasis; Aq, aqueous. Patients whose samples were not measured or with uncertain values (for serum samples) were put in the Uncertain group.

One hundred and eighty aqueous and 207 serum samples, including 172 aqueous and serum pairs were tested for T-IgG. The results are shown in Table 3. The grouping of all participated patients according to their clinical features and aqueous and serum T-IgG status are shown in Figure 2. Six blood samples were not measured because of hemolysis. The results of the 5 samples were not conclusive since the T-IgG values fell into the gray area as defined by the kit. GWC was calculated in all aqueous T-IgG positive samples. The lowest GWC was 6, suggesting the intraocular origin of the antibody.

**Table 3:** Aqueous and serum T-IgG status.

**Category**	**Number of eyes/patients (% in all measured samples in each group)**		
	**Peripheral granuloma**	**Posterior granuloma**	**Endophthalmitis**
Aqueous			
Positive	55 (78.6%)	18 (48.6%)	47 (66.2%)
Negative	15 (21.4%)	19 (51.4%)	26 (33.8%)
Not Measured	13	1	13
Serum			
Positive	56 (70.0%)	15 (28.8%)	44 (52.4%)
Negative	23 (28.8%)	21 (56.8%)	37 (44.0%)
Uncertain	1	1	3
Not Measured	3	1	2
T-IgG consistency			
Both positive	43 (64.2%)	10 (28.6%)	33 (47.1%)
Both negative	10 (14.9%)	13 (37.1%)	21 (30.0%)
Aq+/Serum-	10 (14.9%)	7 (20.0%)	11 (15.7%)
Aq-/Serum+	4 (6.0%)	5 (14.3%)	5 (7.1%)
*Kappa* value	0.454	0.311	0.533

*T-IgG status referred to positive, negative, or uncertain of the anti-T. canis antibody in samples. The definition for positive, negative, and uncertain T-IgG status was described in the Methods. T-IgG consistency referred to the number of patients whose aqueous and serum samples showed the same T-IgG status. + referred to T-IgG positive, – referred to T-IgG negative. Aq, aqueous*.

Overall, 66.7% of the aqueous in this cohort was T-IgG positive. When separated by groups, 78.6, 48.6, and 66.2% of the peripheral granulomatous, posterior granuloma, and endophthalmitis OT eyes were positive, respectively. About 70, 28.8, and 52.4% of the patients were serum T-IgG positive.

In 42 patients (24.4% of all measured pairs), the T-IgG status of the serum and the aqueous of the affected eye were not consistent. There were 28 patients who were aqueous T-IgG positive but serum T-IgG negative and 14 patients who were aqueous T-IgG negative but serum T-IgG positive. To explore the agreement between the aqueous and serum T-IgG, when separated by groups, the kappa values of peripheral granulomatous, posterior granuloma, and endophthalmitis OT eyes were 0.454, 0.311, and 0.533, respectively.

There were 44 patients who were T-IgG negative in both serum and the aqueous samples, suggesting the absence of OT. Their collective clinical features are summarized in Table 4. The posterior granuloma group had the highest percentage of double-negative eyes, followed by the endophthalmitis group and peripheral granuloma group. Overall, the clinical presentations of the double-negative eyes were very similar to those of the entire group. In eyes with peripheral granuloma, the double-negatives had a higher percentage of mild vitritis and a lower percentage of vitreous strands and vitreous veil. In eyes with endophthalmitis, the double-negative had a higher percentage of vitreous strands and vitreous veil.

**Table 4:** Major clinical features of the serum and aqueous T-IgG negative eyes.

**Variables**	**No. of eyes (% in the group)**			
	**Peripheral granuloma**	**Posterior granuloma**	**Endophthal-mitis**	**Overall**
No. of eyes	11	13	20	44
% in the initial group	13.3%	34.2%	23.3%	21.3%
Vitritis				
Severe	4 (36.4%)	3 (23.1%)	10 (50.0%)	17 (38.6%)
Moderate	2 (18.2%)	3 (23.1%)	8 (40.0%)	13 (29.5%)
Mild	5 (45.5%)	7 (53.8%)	2 (10.0%)	14 (31.8%)
Vitreous strands	6 (54.5%)	5 (38.5%)	14 (70.0%)	25 (56.8%)
Vitreous veil	0 (0.0%)	0 (0.0%)	4 (20.0%)	4 (9.1%)
Retinal fibrotic band	6 (54.5%)	12 (92.3%)	7 (35.0%)	25 (56.8%)
Dragged disc	6 (54.5%)	8 (61.5%)	2 (10.0%)	16 (36.4%)
Epiretinal membrane	2 (18.2%)	6 (46.2%)	2 (10.0%)	10 (22.7%)
Retinal detachment				
Partial	3 (27.3%)	3 (23.1%)	3 (15.0%)	9 (20.5%)
Total	2 (18.2%)	0 (0.0%)	6 (30.0%)	8 (18.2%)

### Aqueous Cytokine Profiles

The concentrations of 19 cytokines were measured in 75 aqueous samples taken from patients who had discontinued both topical and systemic treatments for at least 5 days prior to the sampling. During the initial analysis, we separated samples into 4 groups: Group 1, T-IgG negative in both aqueous and serum; Group 2, aqueous negative but serum positive; Group 3, aqueous positive but serum negative; and Group 4, aqueous and serum both positive. We found no significant differences in cytokines between Groups 1 and 2 or between Groups 3 and 4, indicating that T-IgG seropositivity had little effect on aqueous cytokine responses. Therefore, we grouped samples by aqueous T-IgG only: the aqueous T-IgG negative (Aq^−^, 35 samples) and positive (Aq^+^, 40 samples) groups (Table 5). We used 25 aqueous samples taken from eyes with congenital cataract but otherwise quiescent eyes as control. The average age of the children in the control group was 4.5 years, which was significantly younger than the OT patients (unpaired *t*-test, *p* = 0.003).

**Table 5:** Aqueous cytokine concentrations.

**Cytokines**	**Concentrations (average** **±std in pg/mL)**			***p*** **-values**		
	**Cataract**	**Aq-**	**Aq+**	**Aq+/Aq-**	**Aq-/Cat**	**LOD**
	**(*n* = 25)**	**(*n* = 35)**	**(*n* = 40)**			
IL-6	3.3 ± 2.6	23.3 ± 30.6	250.1 ± 383.1	<0.01	<0.01	2.6
MCP-1	158.2 ± 53.6	239.3 ± 164.2	354.8 ± 237.2	<0.01	<0.01	1.1
IL-4	< LOD	< LOD	< LOD			0.7
IL-5	< LOD	0.7 ± 1.1	4.1 ± 5.7	<0.01	0.04	0.6
IL-13	3.2 ± 2.2	0.7 ± 1.2	10.1 ± 12.2	<0.01	<0.01	0.7
IL-10	2.5 ± 1.6	0.7 ± 1	11.2 ± 20.4	<0.01	<0.01	0.3
Eotaxin	< LOD	3.6 ± 1.7	10.5 ± 11.8	<0.01	<0.01	2.5
RANTES	< LOD	< LOD	4.3 ± 9.6	<0.01	0.08	1.8
IL-8	6.3 ± 7.4	13.2 ± 20.1	28.2 ± 30	0.02	<0.01	1.0
TNF-α	< LOD	< LOD	< LOD			6.0
IL-1β	< LOD	< LOD	< LOD			0.6
IL-12p70	8.9 ± 8.7	< LOD	< LOD	<0.01	<0.01	3.5
IFN-γ	10.1 ± 1.6	< LOD	< LOD			6.4
IL-2	< LOD	< LOD	< LOD			1.6
IL-17	< LOD	< LOD	< LOD			3.3
MIP-1α	< LOD	< LOD	< LOD			0.2
MIP-1β	6.0 ± 2.9	4.3 ± 1.5	19.3 ± 18.9	<0.01	0.78	0.8
GCSF	2.8 ± 1.7	4.1 ± 2	8.2 ± 11.9	<0.01	<0.01	1.7
VEGF	52.7 ± 32.6	29.7 ± 26.9	70.4 ± 53.5	<0.01	<0.01	3.1

*Cataract, children with congenital cataract; Aq, aqueous. – and + stood for T-IgG negative or positive in the aqueous, respectively; LOD, limit of the detection as provided by manufacturer*.

Both T-IgG negative and positive groups showed very low concentrations of IL-12 (p70), IFN-γ, IL-2, IL-17, TNF-α, IL-1β, and MIP-1α. The T-IgG positive group (Aq^+^) showed significantly higher IL-6, MCP-1, IL-5, IL-13, Eotaxin, RANTES, IL-8, MIP-1β, and IL-10 than the T-IgG negative group (Aq^−^) and controls. A small yet significant increase of IL-6, MCP-1, IL-8, IL-5, and Eotaxin was observed in the Aq^−^ group compared to controls.

We also compared cytokine concentrations by three clinical types. No statistically significant differences were found among groups (data not shown).

## Discussion

This is probably the largest pediatric OT series reported. Demographically, this cohort of patients recapitulated features reported by previous studies, i.e., boy and rural living environment predominant (6, 10, 11, 14, 21, 24–26). A previous study in China also reported a similar male predominance in OT patients (14). The average age of this cohort was 7.7 years old, which was within the age range with a high seroprevalence of anti-*T. canis* antibody (16, 27). Overall, these demographic features represented factors leading to increased exposure to infectious sources.

We found two clinical features that were not often reported by previous studies. Firstly, the endophthalmitis type constituted 41.5% of the entire cohort. After we excluded patients who were T-IgG antibody-negative in both serum and aqueous, the percentage remained 39.9%. Many studies reported a 25% or less endophthalmitis type in OT (6, 11, 28). However, this number was calculated in cohorts mixed with pediatric and adult patients. There was one study from Poland that reported 48% endophthalmitis type in 94 OT eyes from patients of 3–18 years old, and their diagnosis was based on both clinical presentations and positive serum antibody (8). The ratio of the peripheral granuloma type to the posterior granuloma type in this cohort (2:1) was similar to published data (6, 28).

Another interesting feature of the cohort was the high incidence of retinal detachment (42.2% eyes). In the granuloma types, partial detachment was more frequently seen than total detachment. In the endophthalmitis group, about 83.3% (35 out of 42 eyes) was total detachment. Studies based on patients of all ages in the United States reported <30% retinal detachment in OT eyes (11, 29). In adult OT patients, the retinal detachment was even less frequently seen (10, 24, 30). However, in another study of 35 Chinese OT patients (mostly granulomatous type), a 45.7% retinal detachment was reported (14). We believe that this was largely due to the fact that children were less likely to notice and report vision changes than adults, especially when it occurred only in one eye.

Although aqueous T-IgG positive and negative eyes presented with similar clinical features, they showed different aqueous cytokine profiles. Only aqueous T-IgG positive eyes had increased IL-5, IL-13, IL-10, GCSF, MIP-1β, and RANTES, suggesting a polarized Th2 response. The concentrations of IL-6, MCP-1, IL-8, and Eotaxin were increased in both T-IgG positive and negative eyes, but were higher in the former group. IL-2, IL-4, IFN-γ, TNF-α, IL-12p70, IL-17, and MIP-1α were undetectable in both groups, suggesting the absence of Th1 or Th17 responses.

Interleukin-4 subverts the host tissue immune responses and cell metabolism in favor of parasite survival and growth (31). During the establishment of *T. canis* infection in mice, a synchronized increase of IL-4, IL-5, and IL-13 was found. However, IL-4 was not always increased in serum of patients with systemic *T. canis* infection (3, 4, 32–34). Although IL-4 and IL-13 (or IL-5, which largely tracks with IL-13) are both cytokines central to type-2 inflammation, there do exist some occasions where IL-13 production outweighs IL-4 production in type-2 inflammation. Th2 cells produce IL-13 in peripheral tissues, such as the lung and eye, while IL-4 protein production is centralized to germinal center reactions within B follicles in lymphoid tissues. Since IL-13 drives peripheral hallmarks of type-2 inflammation, IL-13 expression may predominate over IL-4 in non-lymphoid tissues and at mucosal barriers. This phenomenon has been detected in many settings of allergic type-2 lung inflammation (35). As we know, the eye is one of the immunoprivileged organs mainly due to the lack of lymphatic drainage of the retina. Therefore, IL-13 and IL-5 expressions predominate over IL-4 in these none-lymphoid tissues, which may explain why IL-4 was undetectable in our OT groups. Moreover, previous infection and anthelmintic treatment could affect the production of IL-4 or IL-5 (36). Ocular immune responses could be activated by live larva entering the eye or larva dying in the cyst. There were few reports on aqueous cytokine changes associated with OT. Additional analysis, such as the measurement of IgE, could provide more useful information. Unfortunately, there were not enough samples left after T-IgG and cytokine analysis in this study.

Many labs measure T-IgG in serum or intraocular samples to confirm the diagnosis of OT (9–11, 13, 14). Serum samples were much easier to obtain than intraocular fluids. However, the present study showed that aqueous cytokine changes were not associated with serum T-IgG status. Furthermore, the consistency of T-IgG between serum and aqueous samples was about 75%. Therefore, our results suggested that T-IgG should be analyzed in the aqueous instead of serum to provide accurate data on *T. canis* infection.

Negative T-IgG in intraocular fluid samples or serum was often reported in clinically diagnosed OT patients, the percentage of which varied between 15 and 75% in the intraocular fluid and 30 and 87% in the serum (9–11, 13, 14). However, OT cannot be excluded on the basis of negative T-IgG. Factors, such as the low number of larva in the eye, history of the previous infection, insufficient time for the development of immune reactions and antibodies, and the efficiency of the host immune systems could all lead to negative T-IgG (37). Furthermore, the cutoff values for antibody titer of serum and intraocular fluid remain to be further defined. In a most recent study on OT with 290 participants, new diagnostic cutoff values for serum (8.2 U) and intraocular fluid (1.8 U) T-IgG were proposed with increased sensitivity and specificity (38). These cutoff values were lower than what we used in the current study. Using the proposed cutoff values, 3 patients were re-diagnosed as serum T-IgG positive and 1 was re-diagnosed as aqueous T-IgG positive (data not shown). However, the discordant T-IgG titer between aqueous and serum samples of the same patient remained. In the present cohort, there were 11 patients with very low T-IgG in the aqueous (<1 U) and high T-IgG in the serum (> 20 U) and 12 patients with low T-IgG in the serum (<5 U) and high T-IgG in the aqueous (> 10 U).

In this study, most patients were treated with systemic plus topical anti-inflammation medication, depending on the severity of the condition. Anthelmintic medicine was often prescribed. Surgical intervention was given to eyes with vitreous haze and partial retinal detachment. Our treatment strategy was similar to most of the studies published (28, 39). Frequent follow-up was necessary to monitor and adjust treatment strategies.

## Conclusions

In summary, this study provided evidence to suggest the measurement of anti-*T. canis* antibody in aqueous for more specific diagnosis of clinically suspected OT. The analysis of aqueous cytokines could unveil molecular targets for the development of more specific and effective treatment options. However, future studies with fine control of patient history are needed to confirm and further understand the immunological features associated with OT.

## Data Availability Statement

The raw data supporting the conclusions of this article will be made available by the authors, without undue reservation.

## Ethics Statement

The studies involving human participants were reviewed and approved by Institutional Review Board of Xinhua Hospital. Written informed consent to participate in this study was provided by the participants' legal guardian/next of kin. Written informed consent was obtained from the individual(s), and minor(s)' legal guardian/next of kin, for the publication of any potentially identifiable images or data included in this article.

## Author Contributions

Conceptualized, design, and provided fundings for the study: JinL and PZ. Clinical examination and evaluation of the patients: PZ, YH, and XZ. Patient data curation: XZ, YZ, YY, YR, and JiaL. Laboratory investigation: XZ, YR, and JinL. Manuscript writing and editing: JinL, YY, and XZ. All authors contributed to the article and approved the submitted version.

## Funding

This research was supported by grants from the National Natural Science Foundation of China (NFSC) 81873679 to JinL and 82171069 to PZ.

## Conflict of Interest

The authors declare that the research was conducted in the absence of any commercial or financial relationships that could be construed as a potential conflict of interest.

## Publisher's Note

All claims expressed in this article are solely those of the authors and do not necessarily represent those of their affiliated organizations, or those of the publisher, the editors and the reviewers. Any product that may be evaluated in this article, or claim that may be made by its manufacturer, is not guaranteed or endorsed by the publisher.
